# The role of VWF/ADAMTS13 axis in COVID-19-associated coagulopathy

**DOI:** 10.3389/fcvm.2026.1981718

**Published:** 2026-10-13

**Authors:** Quan Zhang, Ye Tan, Meijuan Peng, Shangjie Wu, X. Long Zheng

**Affiliations:** 1 Department of Pulmonary and Critical Care Medicine, The Tenth Affiliated Hospital of Southern Medical University (Dongguan People’s Hospital), Dongguan, Guangdong, China; 2 Departments of Pathology and Laboratory Medicine, The University of Kansas Medical Center, Kansas City, KS, United States; 3 Department of Pulmonary and Critical Care Medicine, Linxiang People’s Hospital, Yueyang, China; 4 Department of Pulmonary and Critical Care Medicine, The Second Xiangya Hospital of Central South University, Changsha, Hunan, China; 5 Institute of Reproductive and Developmental Sciences, The University of Kansas Medical Center, Kansas City, KS, United States

**Keywords:** ADAMTS13, coagulopathy, COVID-19, endothelial injury, microvascular thrombosis, SARS-CoV2, von willebrand factor

## Abstract

Coronavirus disease 2019, which broke out in 2019, is a disease caused by SARS-CoV-2 infection. Although this infection mainly affects the respiratory system, a high incidence of thrombotic events has been observed in severe or critically ill cases and in a considerable number of non-survivors of COVID-19. Although the coagulation dysfunction and hypercoagulability of COVID-19 have been reported in the literature, the specific situation of its coagulation function has not been fully studied. The observation of microvascular thrombosis in COVID-19 patients has attracted our attention to investigate the potential inflammatory endothelial injury or damage. Von Willebrand factor (VWF) and its proteolytic protease, A Disintegrin And Metalloproteinase with ThromboSpondin type 1 motifs, 13 (ADAMTS13), play an important role in maintaining the vascular health and organ perfusion. Therapeutic and prophylactic anticoagulation are important measures to prevent these kinds of thrombotic events, but new treatments such as recombinant ADAMTS13 and anti-VWF nanobody Caplacizumab are being studied *in vitro* for thrombosis with high efficacy. This review focuses on the discussion of the current research progress of the treatment of COVID-19-associated coagulopathy by correcting the imbalance of VWF-ADAMTS13 axis, the existing problems, and the prospects.

## Introduction

Hypercoagulable state and endothelial dysregulation are the hallmark of pathological changes in COVID-19, resulting from infection with SARS-CoV2. Over 70% of fatal cases exhibit coagulopathy (1, 2). Unlike other acute infections or septic shock, where coagulopathy typically emerges later, the COVID-19-associated coagulopathy presents with early-onset of the disease, which includes venous thromboembolism (VTE), pulmonary embolism (PE), ischemic stroke, and other vascular events (3). Even under prophylactic anticoagulation regimes, 15% of patients develop VTE and 30% show arterial events in the initial European studies of critically ill patients (4, 5). However, the reported incidence rate of thrombosis varies among different cohorts. A large US multicenter study reports the VTE rate of only 7.6%, aligning more closely with the non-critically ill populations (6). Another European study of 184 patients in the intensive care unit (ICU) demonstrates 31% of cases with thrombotic complications. Among those, 27% develop PE (7). Meta-analyses confirm that VTE remains the most common and serious complication in all hospitalized patients with COVID-19 (8).

Autopsy study demonstrated the widespread thrombi in pulmonary, renal, hepatic, and small vessels (9), despite traditional anticoagulant therapy was administered. In a landmark histopathological comparison, Ackermann and colleagues demonstrated that a ninefold higher incidence of microvascular thrombosis in the lungs of COVID-19 patients than in that resulted from influenza A-induced acute respiratory distress syndrome (ARDS) (10). Microscopic evaluation revealed widespread endothelial injury along with an abundance of alveolar capillary megakaryocytes. This localized enrichment megakaryocytes may accelerate platelet production, likely explaining why systemic platelet counts often remain relatively normal inpatients with COVID-19. However, the lung megakaryocytes may also function as antigen presenting cells that mount immune responses (11). Extensive microvascular thrombosis results in microcirculatory disturbances and poor outcomes (12). In addition, an autopsy study of 100 adult cases with COVID-19 revealed features consistent with pathological immune activation, such as hyperferritinemia, hemophagocytosis, and disseminated microvascular thrombosis (13). Specifically, platelet-rich microvascular thrombi were observed in the pulmonary, hepatic, and cerebral vessels. Other autopsy studies of COVID-19 have also reported platelet-rich and von Willebrand factor (VWF)-rich microvascular thrombi in multiple organs (14, 15).

The COVID-19-associated coagulopathy differs from the classical thrombotic microangiopathy (TMA), as schistocytes, hemolysis, and severe thrombocytopenia are rarely observed (3). This unique coagulopathy appears to be driven by immune thrombosis wherein activated neutrophils and monocytes interact with platelets and the coagulation cascade, forming intravascular thrombi in both small and large vessels (16). Microvascular thrombotic complications may precipitate ARDS and other organ failure. Immune thrombosis was initially described as an intrinsic effector pathway of innate immunity to contain pathogens (17, 18), which becomes pathogenic when it is dysregulated, creating a self-perpetuating cycle of microvascular thrombosis and inflammation that can culminate in thromboinflammation and overt disseminated intravascular coagulation (DIC) (19, 20). Central to this pathogenic mechanism is the imbalance of VWF and ADAMTS13 (A Disintegrin And Metalloprotease with ThromboSpondin type 1 repeats,13). This imbalance of VWF and ADAMTS13 is not only a biomarker for the disease severity but also a key player in the pathogenesis of immune thrombosis, tissue ischemia, and mortality in patients with COVID-19. This review summarizes the progress made illustrating the role of VWF/ADAMTS13 axis in pathogenesis of COVID-19-associated coagulopathy.

### Von Willebrand factor

In 1926, the Finnish physician Erik von Willebrand investigated a severe bleeding disorder affecting a large family on Föglö Island in the Åland archipelago and published the first comprehensive description of the hereditary hemorrhagic condition that now bears his name. Following its initial description, the disease remained poorly characterized until 1971, when the protein now known as VWF was first detected by immunological methods. Designated at the time as factor VIII (FVIII)-related antigen, this protein was subsequently found to be synthesized by human endothelial cells in culture (21).

Purification of VWF from human plasma was challenging because of its rapid proteolytic degradation. Although initial purification of VWF was achieved in the 1970s, it was only after 1985 that it was cloned and its primary amino acid sequence fully determined, a breakthrough that enabled the subsequent characterization of its complex domain architecture and multifunctional properties (22–26). The historical difficulty in distinguishing FVIII from VWF stemmed from their non-covalent circulating complex; however, later investigations confirmed that they represent structurally and functionally distinct proteins (27). VWF accounts for approximately 99% of the plasma FVIII–VWF complex by mass, with FVIII circulating at a trace concentration (0.3–0.7 nM) nearly entirely bound to VWF. Within this non-covalent biding, VWF acts as a critical carrier protein that prolongs the intravascular half-life of FVIII and protects it from premature proteolytic inactivation (28, 29).

Human *VWF* gene was cloned by two groups of scientists simultaneously and reported in 1985 (22, 24). The *VWF* gene is mapped to the short arm of chromosome 12 (12p13.31), spanning about 178 kb with 52 exons. The entire genomic locus was not fully characterized until 1989 (30). The coding region of 8,439 bp encodes for a 2,813-amino acid prepro-VWF precursor, including a 22-residue signal peptide, a 741-residue propeptide (VWFpp), and a 2,050-residue mature subunit (Figure 1A) (24, 31, 32).

**Figure 1:**
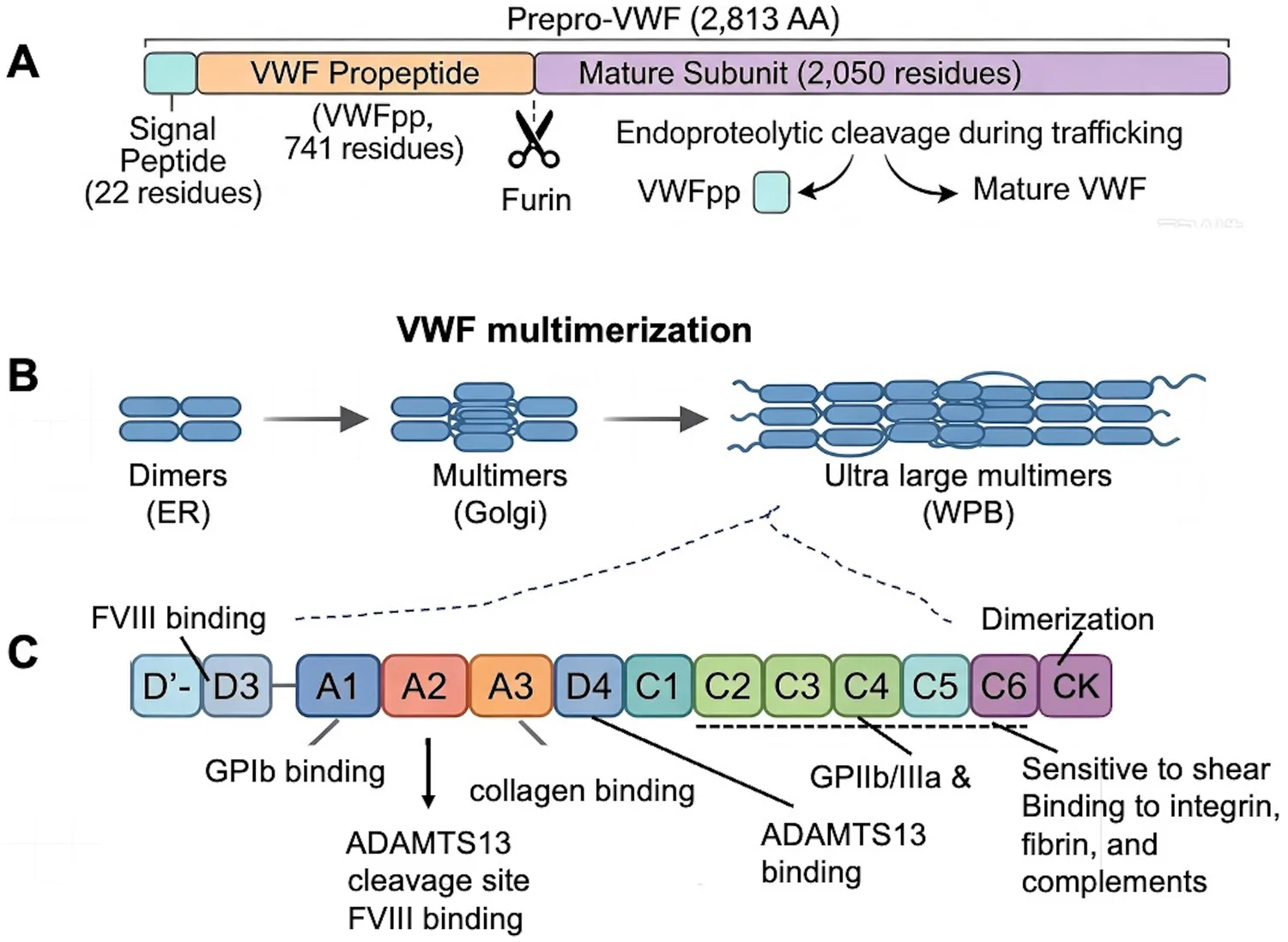
Schematic representation of the human VWF protein precursor, multimerization, and domain interactions. **(A)** Domain organization of the prepro-VWF polypeptide, consisting of a signal peptide, propeptide, furin cleavage site, and mature VWF subunit. **(B)** Assembly of VWF multimers in the endoplasmic reticulum (ER) and Golgi/trans-Golgi network, and storage of ultra large VWF multimers in Weibel-Palade bodies (WPB). **(C)** Functional binding domains of the mature VWF subunit include the D′D3 for FVIII binding, A1 for platelet GP1b binding, A2 for ADAMTS13 cleavage and modulation by ADAMTS13, A3 for collagen binding, D domains for binding to the CUB domains of ADAMTS13 under flow, 6 C domains for shear induced conformational change and binding to integrin, complements, and fibrin, and the CK domain for dimerization.

VWF is produced only in endothelial cells and megakaryocytes. After translation, the prepro-VWF precursor enters the endoplasmic reticulum (ER) and is cleaved at the signal peptide. Guided by its propeptide (VWFpp), monomeric units dimerize in a “tail-to-tail” arrangement via C-terminal disulfide bonds (33–35). These dimers then traffic to the Golgi and trans-Golgi network (TGN), where N-terminal “head-to-head” disulfide bonding drives multimerization into ultra-large VWF (ULVWF) polymers exceeding 20 MDa (Figure 1B). Three closely regulated processes occur during post-translational modification of TGN: i) endoproteolytic cleavage by furin removes the propeptide, leaving a mature VWF molecule that is non-covalently bound to a single Weibel-Palade body (WPB), a process requiring high Ca²⁺ concentration and acidic pH to promote helical tubular condensation and WPB assembly (36, 37); ii) extensive N- and O-linked glycosylation is added, including 12 N-linked and 10 O-linked glycans per subunit; and iii) selective sulfation of N-linked oligosaccharides occurs at Asn384 and Asn468 (38).

Endothelial activation by thrombin, histamine or inflammatory cytokines induces exocytosis of stored WPBs and co-releases ULVWF with VWFpp and angiopoietin-2. Endothelial secretory trafficking originates at the TGN and shows strong apical-basolateral polarity. Regulated secretion of prothrombotic ULVWF occurs at the apical (luminal) cell surface to promote vascular platelet adhesion. In contrast, constitutive secretion of lower-molecular-weight VWF is directed basolaterally to reinforce the subendothelial matrix. In addition, low level basal exocytosis of WPBs from unstimulated endothelium occurs mainly at the apical membrane and is the primary source of steady-state circulating plasma VWF (39).

VWF is a required multi-unit complex that connects hemostasis, thromboinflammation and leukocyte recruitment. After damage to the blood vessel wall, subendothelial VWF binds with exposed collagen to create an adhesive foundation and recruit platelets under high-shear conditions (40–42). VWF can also bind to and protect FVIII from degradation by proteases. Under fluid shear flow, released ULVWF adopts a hyper-reactive multimeric string structure and is transiently bound to the luminal endothelial surface via interactions with P-selectin and integrin *α*v*β*3 (43, 44).

Besides VWF, the WPB secretome includes various vasoactive and immunomodulatory proteins, such as tissue-type plasminogen activator (tPA), P-selectin, interleukin-8 (IL-8), eotaxin-3, angiopoietin-2, osteoprotegerin, endothelin-1, endothelin-converting enzyme, and calcitonin gene-related peptide (45, 46). ULVWF multimers in WPBs are organized into densely packed helical tubular arrays (47). WPB biogenesis is directly regulated by VWF expression; in VWF knockout mice, due to a complete absence of VWF synthesis, mature WPB cannot be formed and agonist-induced P-selectin surface translocation is impaired (48), thereby reducing acute leukocyte recruitment. Therefore, the defect is likely to interfere with the regulatory function of VWF synthesis and storage in inflammation.

A single mature VWF polypeptide is organized from amino terminus to carboxyl terminus as: D'D3-A1A2A3-D4-C1C2C3C4C5C6-CK (Figure 1C) (49, 50). Each of these domains has its unique role in biogenesis and pathophysiological function. For instance, the C-terminal CK (cysteine-knot) domain promotes C-terminal dimerization of VWF polypeptides in the endoplasmic reticulum. The D'D3 domains bind FVIII with high affinity (51, 52). The central A1, A2, and A3 domains have a compact globular fold that coordinates platelet glycoprotein (GP) Ib*α*, ADAMTS13 proteolysis, and collagen binding, respectively. The extended tandem C-terminal VWF modules are relatively flexible, likely to be regulated by shear, which induces conformational change of VWF from a compact, globular state to an extended, biologically active filament in the arterial microcirculation (49). Arterial shear, particularly the turbulent shear, is required to induce VWF conformational change, opening the proteolytic cleavage site (Tyr-Met) for ADAMTS13 (53–55). The proteolytic cleavage of VWF by ADAMTS13 is crucial for regulation of VWF homeostasis and pathophysiological functions.

## ADAMTS13

ADAMTS13, a plasma metalloprotease that cleaves VWF, was first identified and cloned in 2001 (56, 57). The human *ADAMTS13* gene is located at 9q34 and consists of 29 exons (37 kb). The mature transcript (4.7 kb) encodes a 1,427-amino-acid pre-proenzyme. The domain architecture of ADAMTS13 includes a signal peptide (S), a short propeptide (P), a metalloprotease (M) domain, a disintegrin-like (D) domain, the first thrombospondin type 1 repeat (T1), a cysteine-rich (C) domain, a spacer (Spa) domain. The more distal C-terminus contains additional seven TSP1 repeats (T2–T8) and two C-terminal CUB domains (Figure 2) (56, 57). Both the signal peptide and propeptide are cleaved by an intracellular protease before its secretion. ADAMTS13 is primarily synthesized in hepatic stellate cells (58, 59) and endothelial cells (60–62). The secreted ADAMTS13 in plasma binds to ultra large VWF multimers released from activated or injured endothelium via both shear-dependent and shear-independent docking modes (63), resulting in a rapid cleavage of anchored ULVWF polymers on endothelial surface (64). ADAMTS13 cleaves VWF at the specific Tyr^1605^–Met^1606^ bond, which relies on cation ions such as Zn^2+^, Mg^2+^ and Ca^2+^ (54), thus an EDTA-anticoagulant samples are not suitable for ADAMTS13 activity assay. Structure-function study demonstrates that the proteolytic cleavage of peptidyl or multimeric substrate primarily depends on the N-terminal MDTCS domains (65, 66) while the function of more distal C-terminal domains remains elusive. Recent studies show that the distal TSP1 repeats and CUB domains may interact with the N-terminal domains to allosterically inhibit ADAMTS13 activity (67). Low pH, ligand binding or autoantibody (against the distal C-terminal domains) binding appears to disrupt the allosteric inhibition, activate ADAMTS13, and increase ADAMTS13 proteolytic activity (67–70). These findings suggest that proteolytic cleavage of VWF by ADAMTS13 can be regulated at the substrate and enzyme levels.

**Figure 2:**
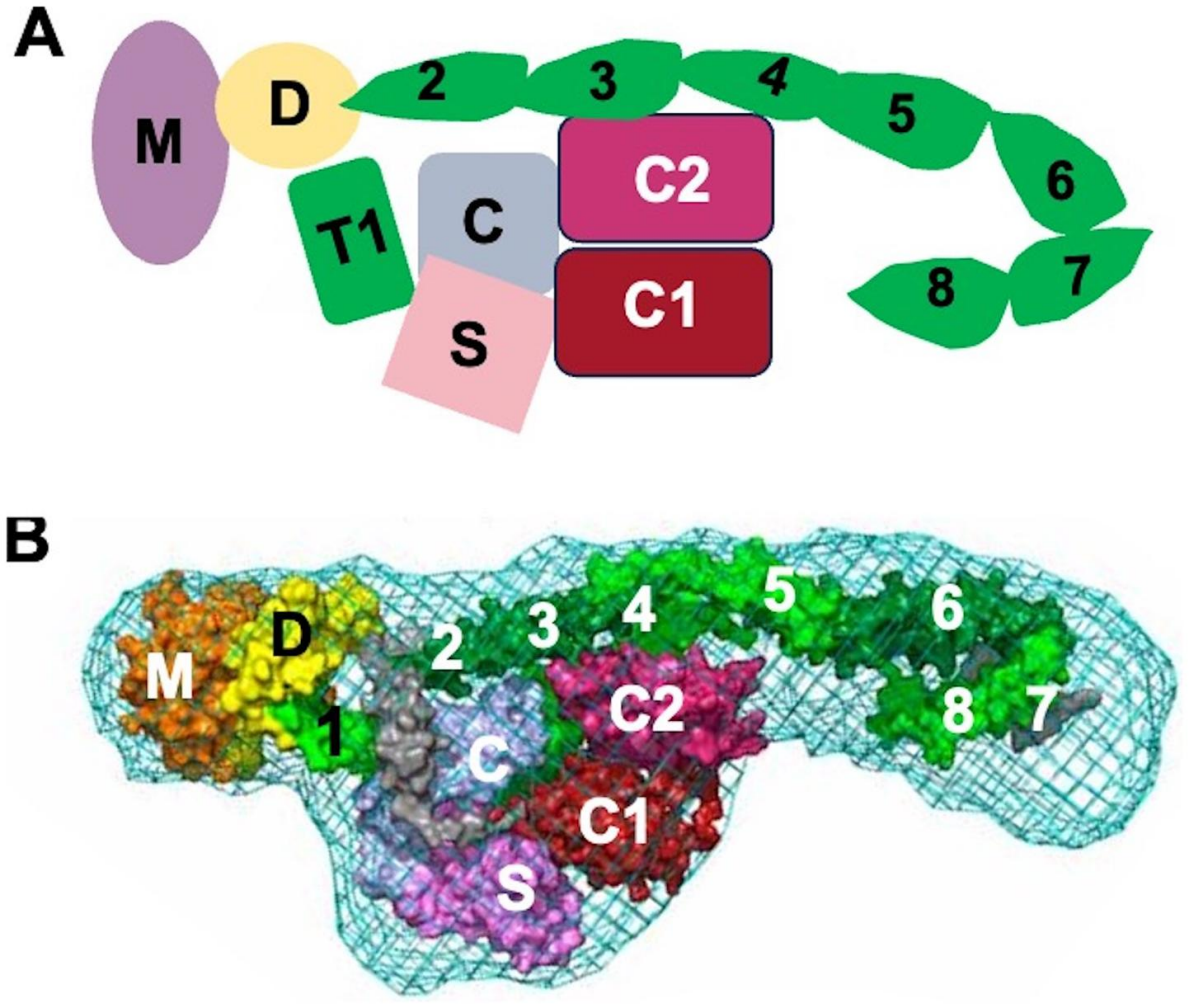
Domain structure and presumptive conformation of ADAMTS13. **(A)** schematic representation of various domains; **(B)** Conformation revealed by small angle x-ray image analysis. Here, M, metalloprotease; D, disintegrin; C, Cysteine-rich domain; T1, the first thrombospondin type (TSP) repeat; S, spacer domain. Number 2–7 are additional TSP repeats; C1 and C2, the first and second complement subcomponents C1r/C1s, Uegf, and bone morphogenetic protein (BMP) 1 domain. (Adapted from Zheng, XL ADAMTS13: structure and function. In: Rodgers, G. (eds). ADAMTS13. Cham: Springer (2015) 39-57. 10.1007/978-3-319-08717-7_3).

## Crosstalk of VWF with leukocytes, NETs, and complements

Inflammation is an organized response of the host to injury or infection in tissues for eliminating harmful factors, removing dead cells, and promoting healing. After recognizing pathogen- or damage-associated molecular patterns (PAMPs or DAMPs), the resident immune cells of the tissue, such as macrophages, mast cells and monocytes release pro-inflammatory cytokines such as interleukin-1 (IL-1), IL-6, and tumor necrosis factor-alpha (TNF-α), etc. These cytokines activate endothelial cells to cause exocytosis of WPBs to rapidly release their cargo proteins, such as VWF and P-selectin, etc. VWF is also known to promote thromboinflammation and hemostasis. Bound VWF directly binds polymorphonuclear neutrophils (PMNs) and monocytes *in vitro* under both static and flow conditions (71–73). The molecules that mediate such an interaction include leukocyte surface receptors, such as low-density lipoprotein receptor-related protein 1 (LRP-1), P-selectin glycoprotein ligand-1 (PSGL-1), β2 integrins, scavenger receptor class A (74), macrophage galectin-3/macrophage galactose-type lectin (75), Siglec-5, and galectins-1 and -3 (76, 77). These molecules all bind to specific domain on the VWF polymer. Additionally, previous studies conducted using animal models of atherosclerosis, wound healing, experimental autoimmune encephalomyelitis, cytokine-induced meningitis and stroke have demonstrated that VWF plays a critical role in inflammation (78–80). Lerolle and his colleagues (81) have shown that in the cecal ligation and puncture (CLP) model of polymicrobial sepsis, v*wf*-null mice exhibit a significantly prolonged lifespan compared to the wild-type mice (81). Hillgruber and others have shown that antibody-mediated VWF neutralization reduces neutrophil infiltration by 50%–65% in murine models of vasculitis and contact dermatitis (82, 83). To rule out confounding defective WPB organelle biogenesis in gene-targeted mice, acute pharmacological VWF blockade in wild-type mice was performed and shew the reduced leukocyte extravasation by about 50%. Petri and others have also found that antibody-mediated inhibition of VWF function reduced the leukocyte recruitment by about 50% in both thioglycolate-induced peritonitis and keratinocyte-derived chemokine (KC)-stimulated cremaster muscle preparations (84). Interestingly, VWF-dependent peritoneal neutrophil recruitment required platelet GPIb*α*, but cutaneous inflammatory recruitment proceeded independent of GPIb*α*. These results suggest tissue- and context-specific mechanobiological pathways.

In a neuroinflammatory state, such as the experimental intracerebral hemorrhage, exogenous VWF administration reduced the lesion accumulation of activated microglia and neutrophils in the area (85). VWF engages macrophage LRP-1 to activate the downstream p38 MAPK and NF-*κ*B signalling pathways, thereby promoting polarization into a pro-inflammatory M1 phenotype (86). In addition to acting as the core for organizing WPB, the released VWF also forms functional post-secretory complexes with co-released WPB proteins (such as galectins, angiopoietin-2 and osteoprotegerin) to enhance the inflammatory signal (87). In short, the above results indicate that VWF is required for blood coagulation, innate immunity, and sepsis-associated thrombotic injury.

More recently, VWF has been found to be required for the construction and stabilization of neutrophil extracellular traps (NETs). Upon activation in acute inflammation, neutrophils release decondensed nuclear chromatin coated with cytotoxic granule proteins, such as neutrophil elastase and myeloperoxidase (MPO), to form NETs (88, 89). Although these web-like fibrillar networks developed as an innate defense against invading pathogens, they also avidly bind circulating VWF and form a prothrombotic scaffold that promotes deep vein thrombosis. Under high shear stress, the VWF–NET axis functions as a strong adhesive platform to promote platelet adhesion, leukocyte recruitment and local accumulation of coagulation factors (90, 91). Grässle has determined that VWF binds to the extracellular DNA backbone of NETs through its A1 domain, and this interaction preserves ADAMTS13-mediated proteolysis of the VWF multimer (92). In murine liver injury models, antibody-mediated VWF blockade or ADAMTS13 cleavage significantly reduces extracellular DNA, neutrophil elastase, and histone deposition without inhibiting the initial recruitment of neutrophils (93).

In addition to endothelium, megakaryocytes and platelets are also the sources of VWF that support this axis. Carestia et al. have found that blocking platelet-derived VWF prevents platelet-induced NETosis, and platelet-derived VWF is required for the activation of both platelets and neutrophils (94). Our group have demonstrated that both NETosis and NET assembly in immune-mediated thrombotic thrombocytopenic purpura (iTTP) are closely associated with VWF and functional platelets (95). Targeted interventions with recombinant ADAMTS13 or caplacizumab (an anti-VWF nanobody) have effectively reduced NET formation and microvascular thrombosis under fluid shear stress. Extracellular DNA and histones in NETs also directly bind to VWF strings to form a self-amplifying feedback loop that promotes platelet aggregation and thrombus expansion (92, 96). The presence of VWF in NETs also results in worsen injury to the myocardium in the ischemia/reperfusion model by increasing inflammatory leukocyte infiltration.

Finally, VWF is a core regulator of the complement cascade by direct and indirect molecular interactions (97–99). Complement factor H (CFH) binds to the VWF A1 and A2 domains and thus competitively inhibits ADAMTS13-mediated cleavage of VWF. At the same time, VWF directly binds to complement components C1q, C3 and C3b, and as a cofactor, promotes complement factor I (CFI)-mediated inactivation of C3b (100). These dynamic connections may play a crucial role for hemostasis, inflammation, thrombopoiesis, and innate immunity.

## The role of VWF/ADAMTS13 in COVID-19-associated endotheliopathy

The main functions of the single layer of vascular endothelium in maintaining systemic vascular homeostasis are as the following: i) to maintain the integrity of the blood-vessel barrier; ii) to regulate vascular tone; iii) to modulate hemostasis; iv) to provide an anti-inflammatory, anti-oxidant, and anti-thrombotic surface; v) to regulate cell proliferation; and finally, vi) to control metabolic exchange of ATP, glucose, and amino acids. Endothelium-derived nitric oxide (NO) is critical for the above homeostatic functions (101, 102). Although SARS-CoV-2 primarily affects the respiratory system, it binds to angiotensin-converting enzyme 2 (ACE2), which is cleaved by host proteases such as furin (103), resulting in extensive damage to blood vessels outside the lungs. Endothelial cells are relatively exposed sentinels that detect pathogen- and damage-associated signals to regulate immune and vascular responses. SARS-CoV-2 infection causes severe endotheliopathy and endotheliitis of the vascular endothelium, resulting in multi-organ failure and serious thrombotic disorders (104, 105). One key feature of COVID-19 pathogenesis is the rapid exocytosis of WPBs, which releases VWF into the bloodstream and promotes hyper-reactive platelet-endothelial cell adhesion and microvascular thrombosis. It remains to be determined whether SARS-CoV-2 directly causes endothelial damage through infection or indirectly by cytokines and both.

High-affinity viral entry is mediated by ACE2 that binds to the SARS-CoV-2 spike protein with high affinity. The binding affinity is 10- to 20-fold higher than to SARS-CoV-1 (106). ACE2 is widely expressed in human vascular endothelium of various organs (107–116). Post-mortem studies of COVID-19 cases showed an increased endothelial ACE2 expression in relation to the alveolar epithelial cells (10), and electron microscopic images of viral particles were identified in the glomerular capillary endothelial cells (117, 118). Direct vascular invasion has been confirmed in human tissue panels (115, 119–121) and severe transgenic mouse models (e.g., K18-hACE2), which exhibit alveolar capillary infection, local complement deposition and immune cell recruitment (122). Other entry mechanisms include co-receptors such as TMPRSS2, cathepsin L1, neuropilin-1 and CD209L/L-SIGN (123, 124). Endothelial ACE2 expression increases with age through IL-7/NF-*κ*B signaling (125), consistent with the increased susceptibility of severe COVID-19 in elderly population. Analysis of patient thrombi further revealed the increase of endothelial damage markers and formation of NETs (126). These findings support the direct endothelial damage hypothesis causing endotheliopathy.

Several other studies have reported no direct infection of SARS-CoV2 in endothelium. Endothelial-specific hACE2 knockout models (Tie2-Cre) showed vasculopathy like that in wild-type mice. Authors concluded that direct SARS-CoV2 endothelial entry is not required for vasculopathy (127). *In vitro* exposure of human umbilical vein (HUVEC), pulmonary and cardiac endothelial cells (ECs) to SARS-CoV-2 resulted in a non-productive and abortive infection without virion shedding (128, 129). Only after recombinant ACE2 transduction was the permissiveness to viral replication restored (130). Microfluidic organ-on-a-chip and plasma transfusion models have shown that paracrine crosstalk, where infected epithelial cells release proinflammatory cytokines and viral components, is sufficient to disrupt the endothelial barrier function of adjacent cells, cause glycocalyx shedding, and induce hypercoagulability without direct endothelial infection (131–133). Thus, the exact mechanism underlying SARS-CoV2 induced- endotheliopathy remains to be investigated.

Clinical studies, including the data from our group, have shown that marks reflecting endothelial activation and injury and platelet activation are significantly increased in patients with severe COVID-19. These include elevated plasma levels of VWF antigen, soluble P-selectin, plasminogen activator inhibitor (PAI)-1, syndecan-1 (indicating glycocalyx degradation), complement activation fragments and soluble thrombomodulin. The shedding or release of membrane bound vasoprotective thrombomodulin is associated with microvascular damage. Inflammatory endothelial activation also causes a large-scale release of tissue plasminogen activator and PAI-1, and thus provides a mechanism for the substantial increase in D-dimer and fibrin degradation products in COVID-19-associated coagulopathy. Autopsy and clinical series have shown that pulmonary microvascular thrombosis and endothelial injury directly cause the physiological dead space and right-to-left shunting in critically ill patients (134).

Patients with COVID-19 consistently present with the increased levels of VWF antigen and activity with a corresponding reduction in plasma ADAMTS13 activity (9, 135). The increased ratio of VWF/ADAMTS13 is associated with more severe COVID-19 disease and increased all-cause mortality. Non-survivors have more significantly reduced plasma ADAMTS13 activity and elevated plasma VWF antigen than the survivors, and the plasma level of ADAMTS13 activity is inversely related to D-dimer, fibrinogen, and VWF antigen (136). However, interpretation of these observational cohorts should be done with caution due to different sampling times at different stages of the disease and different assay methods.

The specific mechanisms underlying ADAMTS13 deficiency in severe COVID-19 are not fully understood. A system of hypoxia and inflammation activate endothelial cells and trigger WPB exocytosis, which results in massive release of VWF. In the meantime, VWF proteolysis by ADAMTS13 is suppressed via several independent pathways: 1) ADAMTS13 rapidly binds to the excessive substrate VWF accelerating the ADAMTS13 protease consumption and clearance (81); 2) ADAMTS13 may be degraded by active coagulation proteases (e.g., thrombin and plasmin) (137), or inactivated by oxidative modification or reactive oxygen species (ROS) and circulating proinflammatory cytokines (138). Hyperferritinemia has been linked to increased VWF secretion and relative deficiency of ADAMTS13, thus promoting immune-mediated thrombosis and end-organ damage; 3) ADAMTS13 synthesis in hepatic stellate cells and endothelial cells may be suppressed by COVID-19-induced liver injury and circulating systemic cytokines (e.g., IFN-γ, IL-4, and TNF-α), which directly inhibits ADAMTS13 expression and secretion, similar to the patterns observed in acute liver failure and cirrhosis (58, 139); 4) Adrian and his colleagues have found that 34.4% of COVID-19 patients have acquired inhibitory anti-ADAMTS13 autoantibodies that strongly correlate with reduced enzyme activity and an unfavorable prognosis (140). In addition, work by our group and others shows that ADAMTS13 switches to a compact, “closed” non-hyperactive state during severe COVID-19, thus exhibiting an autoinhibition of plasma ADAMTS13 function (Figure 3) (141).

**Figure 3:**
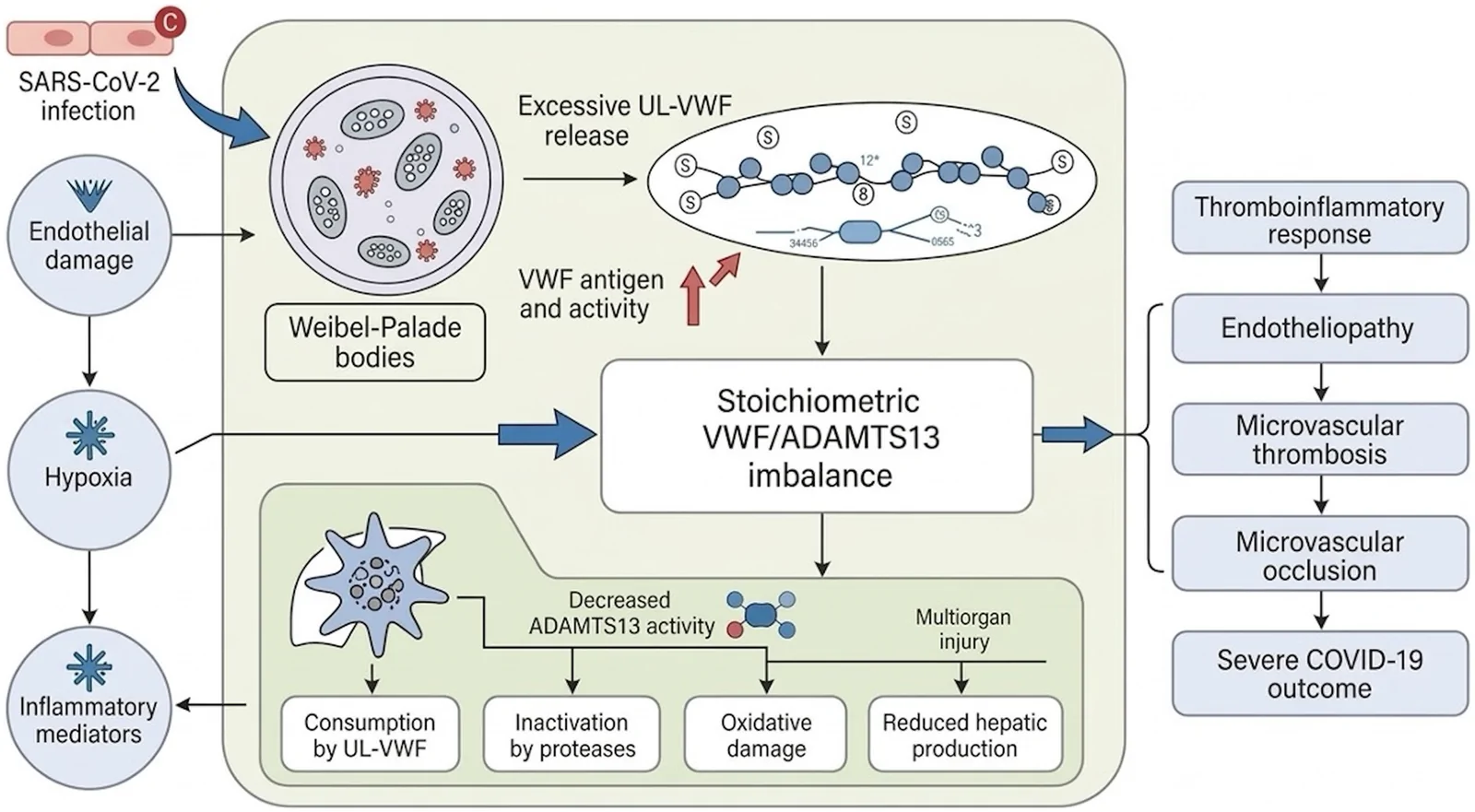
Mechanistic model of the VWF/ADAMTS13 imbalance underlying COVID-19-associated endotheliopathy. SASR-CoV2 directly or indirectly causes endothelial damage, resulting in hypoxia and the release of inflammatory mediators. This triggers the release of VWF from Weibel-Palade bodies and inhibits synthesis of ADAMTS13 from hepatic stellate cells. In the meantime, oxidative stress blocks the proteolytic cleavage of VWF or inactivation of ADAMTS13 enzyme. This results in imbalance of VWF/ADAMTS13, leading to microvascular thrombosis and endotheliopathy in patients with severe COVID-19.

Neutrophil activation and NETosis also regulate the VWF-ADAMTS13 axis to some extent, beyond direct enzyme inhibition. Neutrophil extracellular trap (NET) components are post-translationally modified through oxidation, citrullination and enzymatic sialylation, and these modifications physically block or sterically hinder the molecular interaction between VWF and ADAMTS13 (142–144). When proteolysis is inhibited, stable UL-VWF multimers remain in circulation as highly thrombogenic intravascular strands under high-shear conditions and activate platelets directly, causing pulmonary microvascular thrombosis.

In summary, the COVID-19-associated endotheliopathy is caused by multiple factors and processes: 1) the direct damage and pyroptosis of endothelial cells; 2) shedding of endothelial glycocalyx; 3) endothelial-to-mesenchymal transition (EndMT); 4) vascular inflammation and leukocyte adhesion; 5) elevated oxidative stress and uncoupled NO synthesis; 6) cytokine storm amplification; 7) mitochondrial dysregulation; and finally 8) systemic complement activation (145).

## Targeting VWF/ADAMTS13 as a novel therapy for COVID-19-associated coagulopathy

The extensive body of research reviewed herein demonstrates that COVID-19-associated microvascular thrombosis and multi-organ dysfunction stem from a convergent thromboinflammatory network spanning systemic inflammation, endothelial injury, platelet activation, and severe hypercoagulation. Although population-wide vaccination and prior infection have conferred substantial immunity that significantly mitigates thrombotic risk, the pathophysiological potential of emerging variants remains uncertain. High-risk cohorts, particularly immunocompromised individuals and patients with underlying cardiometabolic comorbidities remain vulnerable to critical illness and severe endotheliopathy.

Endothelial activation causes the widespread exocytosis of ULVWF multimers that exceeds the cleavage capacity of ADAMTS13 and thus leads to platelet tethering and intravascular thrombosis. Preclinical and *ex vivo* studies have identified several effective interventions in this direction for COVID-19. Caplacizumab (an anti-VWF nanobody) (96, 146), anfibatide (a platelet GPIb*α* inhibitor) (147) or CA1001 (a platelet antibody fragment) (148), and recombinant ADAMTS13 supplementation (96) have shown to suppress the NET formation and inhibit microvascular thrombosis in whole blood of COVID-19 and iTTP patient samples. These novel targeted drugs are shown to significantly inhibit platelet adhesion and aggregation and thrombus formation in various animal models *ex vivo* and *in vivo* (96, 147–149). In addition, N-acetylcysteine (NAC) can directly break down ULVWF multimers through a non-enzymatic cleavage mechanism by reducing disulfide bonds in the VWF A1 domain; thus, it inhibits VWF-dependent platelet aggregation and leukocyte recruitment and exhibits thrombolytic activity (150–152).

Beyond these mechanistic findings, clinical evidence for targeting the VWF-ADAMTS13 axis in COVID-19 cohorts is accumulating. NAC treatment was associated with lower mortality (RR 0.65, 95% CI 0.56–0.75) and significant reductions in D-dimer and CRP levels in a meta-analysis of 20,503 patients with COVID-19 (153). Furthermore, NAC supplementation normalized plasma VWF levels and improved symptoms in patients with Long COVID (154). Caplacizumab has shown efficacy in COVID-19-associated TTP in case series, achieving complete hematologic recovery when combined with plasma exchange and steroids (155). Recombinant ADAMTS13 reduces abnormally elevated VWF activity and depletes ULVWF multimers in plasma from severe COVID-19 patients *ex vivo*, in a time and concentration dependent manner (156). In contrast, the P-selectin inhibitor crizanlizumab failed to improve organ support–free days despite reducing soluble P-selectin levels by 89% (157, 158), suggesting that targeting a single downstream adhesion molecule is insufficient to reverse the thromboinflammatory network in CAC.

In short, the above mechanisms and clinical evidence suggest that pharmacologically targeting the VWF-ADAMTS13 axis may be an effective way to reduce microvascular thrombosis and multi-organ damage in COVID-19 and other thromboinflammatory diseases.
